# Molecular Diversification of the Genus *Clinopodium* (Lamiaceae) from the Balkans with an Emphasis on the Transferred Groups *Calamintha*, *Acinos*, and the Sect. *Pseudomelissa*

**DOI:** 10.3390/plants14182940

**Published:** 2025-09-22

**Authors:** Smiljana Janković, Tanja Dodoš, Petar D. Marin, Jelica Obradović Novaković, Nemanja Rajčević

**Affiliations:** Institute of Botany and Botanical Garden “Jevremovac”, Faculty of Biology, University of Belgrade, 43 Takovska Street, 11 000 Belgrade, Serbia; tanjadodos@bio.bg.ac.rs (T.D.); pdmarin@bio.bg.ac.rs (P.D.M.); jelica@bio.bg.ac.rs (J.O.N.); nemanja@bio.bg.ac.rs (N.R.)

**Keywords:** *Acinos*, Balkans, *Calamintha*, Lamiaceae, *Micromeria*, phylogenetic analysis, diversification

## Abstract

*Clinopodium* is a morphologically highly diverse and taxonomically intricate genus. Molecular studies have demonstrated high divergence within the genus, and there is no consensus on the taxonomic treatment of some groups classified as *Clinopodium*. The current phylogenetic understanding of the genus relies almost exclusively on the analysis of the *trn*K and *trn*L-*trn*F sequences. In *Clinopodium s.s.*, there is no phylogenetic backbone based on nuclear sequences. Therefore, in this study, we included a larger number of plastid and nuclear markers to better understand the diversification of natural populations of the genus *Clinopodium* from the Balkans. We encompassed the wild-growing taxa from former genera that have now been integrated into *Clinopodium*: *Calamintha*, *Acinos*, and section *Pseudomelissa* from the genus *Micromeria*. The markers that displayed the highest informativeness in the in silico analysis were selected. Four nuclear loci (ITS1, 5.8S rDNA, ITS2, ETS) and seven plastid loci (*rps*16-*trn*K^UUU^, *rpl*32-*trn*L^UAG^, *rps*15-*ycf*1, *psb*A-*trn*H^GUG^, *rps*16-*trn*Q^UUG^, *pet*N-*psb*M, *psb*K-*trn*S^UGA^) were used to analyse the phylogenetic relationships between the Balkan species and subspecies currently classified into *Clinopodium*. Phylogenetic reconstructions showed the divergence of the two lineages with different diversification patterns. Nuclear markers have shown that the three groups within the clade *Clinopodium s.s.* have evolved separately, which is consistent with earlier phenetic systems.

## 1. Introduction

With over 7000 species, the Lamiaceae family is of great economic and ecological importance [1]. The species of this family are used as pharmaceuticals, food preservatives, spices [2], natural insect repellents [3], as well as ornamentals [4]. Because of its widespread utilisation, fundamental and systematic studies of representatives of this family are crucial and more necessary than ever. Understanding the relationships between the taxa in conjunction with phytochemical data can help with plans for sustainable biological utilisation and biodiversity conservation. Phylogenetically related taxa tend to have more similar biosynthetic pathways and metabolite profiles, so research toward understanding the phylogenetic relationships of this group deserves a high priority.

Molecular profiling is essential when considering the taxonomically complex genera that comprise morphologically and phytochemically diverse species. One such genus is *Clinopodium* L. When first described in Species Plantarum by Carl Linnaeus (IPNI, https://www.ipni.org/p/1071-2, accessed on 20 March 2025), this genus encompassed only three species: *C. vulgare* L., *C. incanum* L., and *C. rugosum* L. Later, *C. incanum* was transferred to the genus *Pycnanthemum* Michx. and *C. rugosum* to the genus *Hyptis* Jacq. Both transferred species are native to the southeastern part of North America, while *C. vulgare*, the species that has kept the taxonomic name since Linnaeus, has a wide natural range, from Europe to Siberia, with an allochthonous distribution in America (POWO, https://powo.science.kew.org/taxon/urn:lsid:ipni.org:names:61206-2, accessed on 13 January 2025). This implies that the initial view of *Clinopodium* was that it was a genus with a mainly Holarctic distribution. *C. vulgare* has been chiefly regarded as the typical species of this genus since its classification has changed considerably from the first description.

Most currently accepted *Clinopodium* taxa are part of the informal *Satureja s.l.* group, specifically of two tribes, Satureineae and Melissinaeae sensu Bentham [5]. A decade later, Bentham [5] reorganised its tribal system, and following this system, taxa *Satureia* L., *Micromeria* Benth., *Calamintha* Moench et. auct., and *Melissa* Tourn. ex L. were defined as separated genera. This group’s most complex and diverse genus was *Calamintha*, which the author separated into five sections: *Calamintha*, *Calomelissa*, *Acinos*, *Clinopodium*, and *Heteromelysson*. The section *Pseudomelissa* Benth. was defined as a part of the genus *Micromeria*. Prominent botanists who agreed with this view and continued research based on the clear separation between these genera were also Boissier [6], Visiani [7], Šilić [8], and Marin [9]. Briquet [10] and Kuntze presented their ideas of the unique genus comprising all the mentioned taxa, where Briquet proposed the name *Satureia* L. and Kuntze *Clinopodium* L.

Despite all the taxonomical problems with the delimitation of the genus *Clinopodium*, its robust position in the family Lamiaceae is clear. In the past three decades, all taxa now belonging to *Clinopodium* have been placed in the subfamily Nepetoideae (Dumort.) Luerss., of the tribe Mentheae Dumort. and the subtribe Menthinae (Dumort.) Endl. [11,12]. However, despite numerous studies, the delimitation of the genus, relationships at the species level, and what falls under *Clinopodium* remain unclear. According to the current classification, *Clinopodium* L. comprises 190 species (POWO, https://powo.science.kew.org/taxon/urn:lsid:ipni.org:names:30008690-2, accessed on 13 January 2025).

Doroszenko [13] conducted a comprehensive review of the *Satureja s.l.* complex, analysing the considerations of his predecessors. Based on his view, the genera *Calamintha* Miller, *Acinos* Miller, *Clinopodium* L., *Cyclotrichium* Manden, and Scheng are separate genera. Nevertheless, they were all loosely classified as “Calaminthoid genera,” primarily adhering to Bentham’s classification. Also, this author divided the genera from *Satureja s.l.* into four groups organized into two clusters: “Calaminthoid” and “New World” (*Gardoguia* Ruiz and Pav., *Obtegomeria* sensu Doroszenko, *Montereya* sensu Doroszenko, *Piloblephis* Rafin., *Hesperothymus* sensu Doroszenko, *Xenopoma* Willd., *Diodeilis* Rafin.). This was a form of geographical delimitation for this group. To examine whether this division is phylogenetically supported, Cantino and Wagstaff [14] conducted a molecular study based on chloroplast fragment length polymorphisms. They concluded that “Calaminthoid” and “New World” taxa sensu Doroszenko form one clade, with some of the other genera from the American continent. Considering morphological characteristics, pollination biology, and the molecular data obtained, they proposed a Clinopodioid complex that includes ten genera. They excluded certain genera from this complex, despite being in a clade with other Clinopodioid genera, due to significant morphological differences between them. Provisionally, they divided the complex into five genera: *Cyclotrichium* (Boiss.) Manden. and Scheng., *Obtegomeria* Doroszenko and P.D.Cantino, *Gardoquia* Ruiz and Pav., *Xenopoma* Willd. (including *Hesperothymus* sensu Doroszenko), and *Clinopodium* (including *Calamintha*, *Diodeilis* Raf., and *Montereya* sensu Doroszenko) [13]. Two years later, Harley and Paucar [15] published a list of about 60 taxa from Tropical America transferred from *Gardoqia* into *Clinopodium*. The authors noted that they followed Doroszenko’s view of *Clinopodium*, although Doroszenko omitted *Clinopodium* in the New World group. More recent molecular studies at the subfamilial and tribal levels of the family Lamiaceae also showed segregation of the taxa from the Clinopodioid genera sensu Cantino and Wagstaff [16,17].

In a parsimony analysis of *trn*K intron sequence data, the section *Pseudomelissa* from the genus *Micromeria* was grouped in a clade with Old World *Clinopodium*, which resulted in the definite transfer of *Pseudomelissa* to *Clinopodium* [18]. *Clinopodium* was additionally expanded by transferring African taxa from the genus *Satureja*, which were named *Clinopodium abyssinicum* and *Clinopodium simense* groups [19,20]. After this, a comprehensive study of the subtribe Menthinae [21] revealed some patterns of diversification among the taxa considered *Clinopodium*, and the very complex status of the genus was shown again. The analysis of *trn*K intron, *trn*L-*trn*F, and ITS sequences showed that the subtribe Menthinae consisted of three clear groups: “*Clinopodium*”, “*Micromeria*”, and “*Satureja*” groups. The *Clinopodium* group, comprising both New and Old World taxa, showed an extraordinary diversity, with as many as 17 subclusters. Especially considering *Clinopodium* taxa with distribution in the Balkans, which are the main interest of this study, it is evident that these taxa form two clades: “*Acinos*”*/Ziziphora* group and *Clinopodium* s.str. Although the authors included many taxa, they noted that a precise delimitation of the genus is not feasible. According to previous investigations and considering today’s nomenclature system, all of the mentioned taxa—*Calamintha* Miller, *Acinos* Miller, section *Pseudomelissa* Benth. from genus *Micromeria* Benth., and *Clinopodium* L. itself—fall under *Clinopodium*.

Following the previous chemotaxonomic analysis of these taxa from the Balkans [22], where former *Acinos* taxa separated strongly from other *Clinopodium* taxa, this study aimed to investigate the phylogenetic relationships of the taxa in the genus *Clinopodium* distributed in the Balkans. The Balkan Peninsula, a geographical region, is recognised as one of the centres of European biodiversity, featuring numerous refugial and diverse habitats while demonstrating relatively high environmental stability over long periods [23]. Thus, this area represents a good natural unit for examining the diversification of genera. In addition, there is a considerable diversity of *Clinopodium* species in the Balkans, including endemic species, area boundaries of particular species (e.g., *C. thymifolium*), but also more widespread species.

Summarizing the available literature [8,24], the estimated number of species of the genus *Clinopodium* in the Balkans is between 25 and 30. When including the infraspecific taxa, this number is somewhat higher. However, the biggest taxonomic problem related to the genus is the inconsistent nomenclature and the actual understanding of the genus. As mentioned earlier, previous studies have considered some *Clinopodium* taxa from the Balkans in the broader context of the subfamily Nepetoideae, but without providing detailed insights into interspecific relationships.

This is the first in-depth insight into the *Clinopodium* sensu stricto clade, as the relationships between the taxa in this clade based on nrDNA were previously unknown. We attempted to convey as much diversity as possible by combining taxa from previously separated genera, now transferred to the genus *Clinopodium*. We combined a set of molecular markers from both nuclear and chloroplast genomes. Three nrDNA spacers: ITS1, ITS2, ETS, one coding region 5.8S rDNA, and seven chloroplast intergenic spacers: *rps*16-*trn*K^UUU^, *rpl*32-*trn*L^UAG^, *rps*15-*ycf*1, *psb*A-*trn*H^GUG^, *rps*16-*trn*Q^UUG^, *pet*N-*psb*M, and *psb*K-*trn*S^UGA^, were used. Most of these loci were used for the first time in this group.

## 2. Results

### 2.1. DNA Regions and Alignments

The sequences for four nuclear and seven chloroplast regions were obtained from fourteen *Clinopodium* taxa, while species from the genera *Micromeria* and *Ziziphora* were used as outgroup taxa. Table 1 shows the informativeness of all the analysed regions, including the number of indels, substitutions, and microsatellites. The final alignment of all nuclear and plastid sequences was 6520 nucleotides long.

### 2.2. Phylogenetic Reconstructions

**Figure 1:**
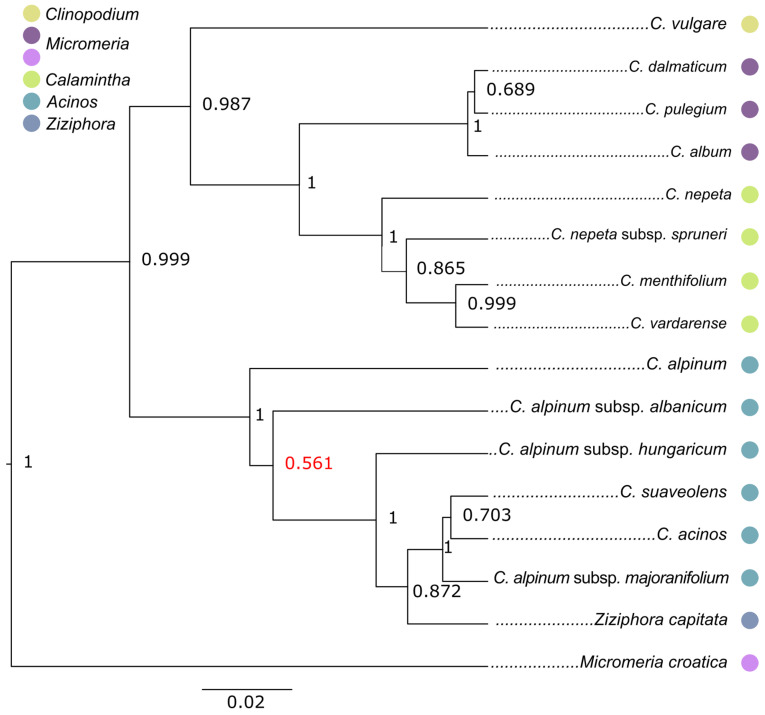
The majority-rule Bayesian tree based on 1110 nucleotides of the ETS, ITS1, 5.8S rDNA, and ITS2 with 14 *Clinopodium* accessions and two outgroups (*Ziziphora capitata* and *Micromeria croatica*). The numbers at nodes are posterior probabilities. The dots next to the names correspond to previous taxonomic treatment of the taxa.

A phylogenetic reconstruction based on nuclear sequences (nrDNA) showed the separation of two clades. This dendrogram was obtained by Bayesian analysis, where the separation of the two primary clades was strongly supported (0.99). The first clade comprised all earlier recognised *Acinos* taxa together with the species *Ziziphora capitata* (*Acinos*/*Ziziphora* clade). The second clade included *C. vulgare*, all previous *Pseudomelissa* taxa, and *Calamintha* taxa (*Clinopodium*–*Pseudomelissa*–*Calamintha* clade). In this clade, groups previously defined by classical systematics received support. One subclade consisted of the former *Pseudomelissa* taxa, while the other comprised the former *Calamintha* taxa. *C vulgare* formed its own subclade (Figure 1).

**Figure 2:**
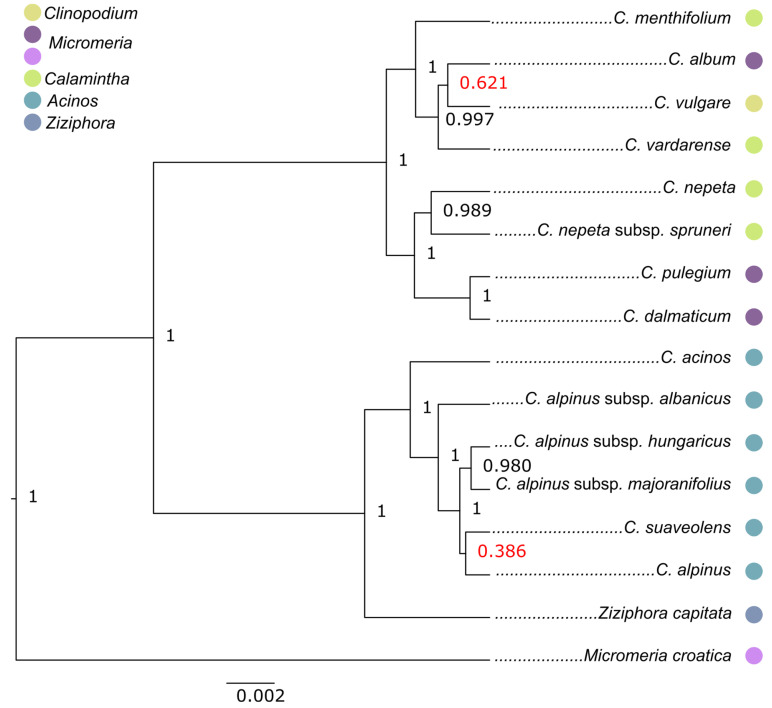
The majority-rule Bayesian tree based on 5410 nucleotides of seven cpDNA regions (*rps*16-*trn*K^UUU^, *rpl*32-*trn*L^UAG^, *rps*15-*ycf*1, *psb*A-*trn*H^GUG^, *rps*16*-trn*Q, *pet*N*-psb*M, *psb*K-*trn*S^UGA^) with 14 *Clinopodium* accessions and two outgroups (*Ziziphora capitata* and *Micromeria croatica*). The numbers at nodes are posterior probabilities, with the nodes with support less than 0.7 marked in red. The dots next to the names correspond to previous taxonomic treatment of the taxa.

The Bayesian analysis of seven chloroplast sequences (cpDNA) revealed a similar cladogram backbone. The genus is divided into the *Acinos*/*Ziziphora* clade and the *Clinopodium*–*Pseudomelissa*–*Calamintha* clade, both well supported (0.99). The positions of the taxa in the subclades changed slightly when compared with the nrDNA data. In the *Acinos*/*Ziziphora* clade, *Z. capitata* is well supported as a sister taxon to all former *Acinos* taxa that together form the subclade. In the *Clinopodium*–*Pseudomelissa*–*Calamintha* clade, there is no clear separation between the formerly well-defined group according to phenetics, but two subclades containing mixed former *Calamintha* and *Pseudomelissa* taxa appear (Figure 2).

**Figure 3:**
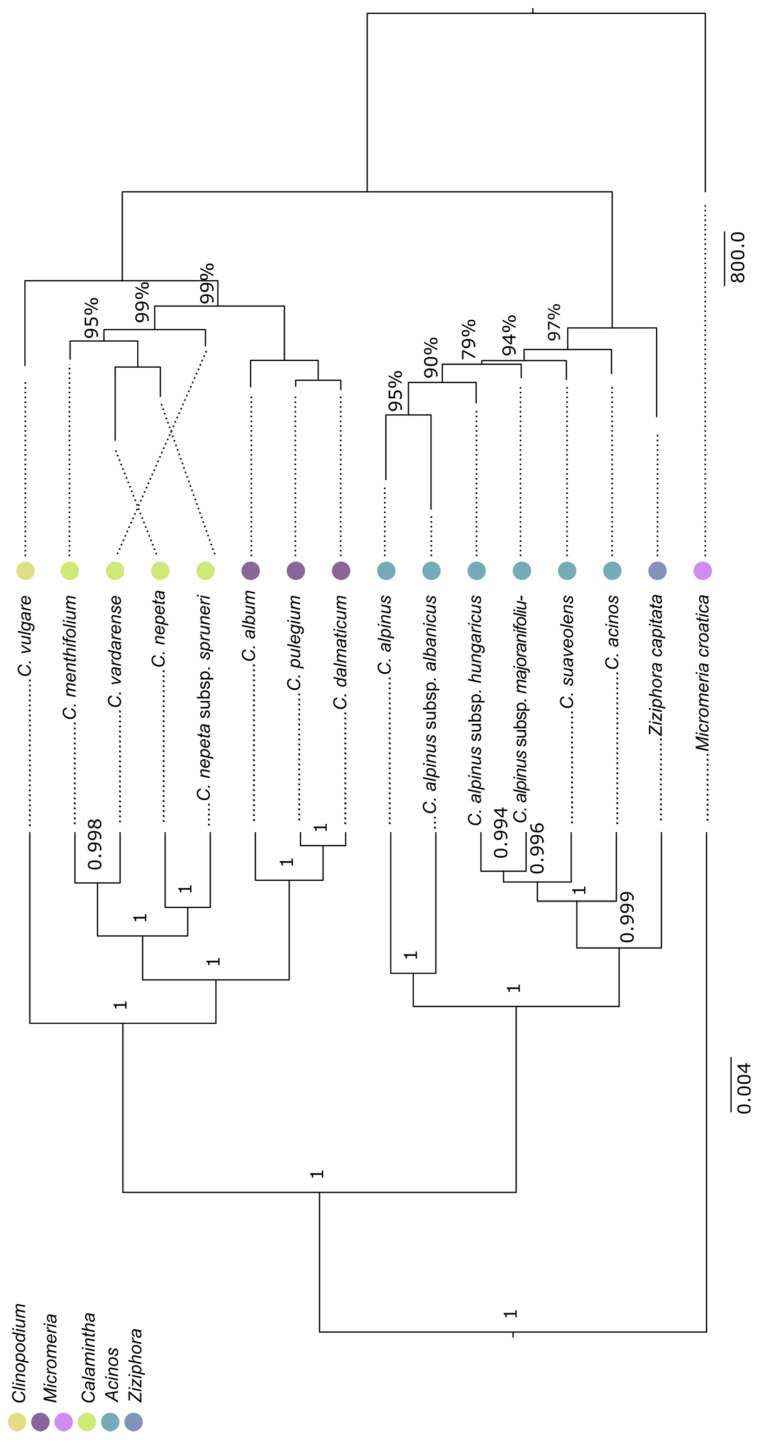
The majority-rule Bayesian tree (left) and the Maximum likelihood tree (right) are based on 6520 nucleotides of four nrDNA and seven cpDNA regions with 14 *Clinopodium* accessions and two outgroups (*Ziziphora capitata* and *Micromeria croatica*). The numbers at nodes are posterior probabilities (left). The numbers above the branches in the tree to the right are bootstrap values and are not provided for branches with a BS value of 100%. The dots next to the names correspond to previous taxonomic treatment of the taxa.

The dendrogram obtained from the combined analysis of the nuclear and chloroplast datasets exhibited a very similar phylogenetic backbone to the previous two. Additionally, due to longer sequences with a higher number of polymorphic informative sites, all of the clades that had the lower statistical support are now missing, and the taxa in the concatenated tree have a different position. This tree largely supports the previous classical systematics of this group. In particular, it enhances the resolution of taxa delimitation within the clade *Clinopodium*–*Pseudomelissa*–*Calamintha*. Conversely, the species *Z. capitata* was not positioned as a sister taxon to the former *Acinos* taxa but between the *Acinos* taxa (Figure 3). The number of parsimony-informative sites (PISs) separating the *Acinos*/*Ziziphora* clade from the other clade is different for each region: *trn*S-*psb*K—4, *trn*L-*rpl*32—22, *rps*16-*trn*Q—21, *rps*16-*trn*K—10, *rps*15-*ycf*1—3, *psb*A-*trn*H—7, *pet*N-*psb*M—2, and ITS—12. In the ETS region, PISs common to all taxa in the *Acinos*/*Ziziphora* clade were not detected.

## 3. Discussion

### 3.1. Acinos/Ziziphora Clade

When considering the rough position of the genus *Clinopodium* in the family Lamiaceae, several studies showed that this genus was generally positioned on a common clade consisting of the “Clinopodioid complex” sensu Cantino and Wagstaff, together with the other genera distributed on the American continent [14,16,17]. The “Clinopodioid complex” is proposed for the first time in the study by Cantino and Wagstaff [14] and comprises ten genera: *Acinos*, *Calamintha*, *Diodelis*, *Gardoquia*, *Hesperothymus*, *Montereya* sensu Doroszenko, *Clinopodium*, *Cyclotrichium*, *Obtegomeria*, and *Xenopoma*. Essentially, this group is a combination of “New World genera” and “Calaminthoid genera” (Old World taxa) sensu Doroszenko. Cantino and Wagstaff [14] combined both phenetic and phylogenetic approaches to define the “Clinopodioid complex,” as they believed that some genera should not be included in the complex due to the differing morphology between the taxa within the obtained clade. More recent studies [16,17] have also supported similar segregation. Combining six chloroplast protein-coding sequences, Li et al. [16] supported the rough position of the genus *Clinopodium* in the tribe Mentheae, but only *C*. *vulgare* L. was included in the study. In the mentioned research, the closest sister group of *C. vulgare* was *Ziziphora* (represented by the species *Z. taurica* M. Bieb), and the other sister clade is composed mainly of New World taxa. Following this study, Zhao et al. [17] conducted a much more extensive study looking at 79 protein-coding plastid genes to understand the relationships at the tribal level in the family Lamiaceae. Based on this comprehensive research, *Clinopodium abyssinicum* (Benth) Kuntze (distributed in Tropical Africa and the Arabian Peninsula) is also in the subclade with the American taxa in the subtribe Menthinae. *C. abyssinicum* is a sister taxon of *Poliomintha bustamanta* B. L. Turner, whose other sister groups are the genera *Dicerandra* Benth., *Monarda* L., and *Pycnanthemum* (genera with American distribution). The phylogenetic position of *C. vulgar*e and *C. abyssinicum* close to New World taxa in the same clade makes it further challenging to define generic boundaries. The main problems in delimiting the genus are the unresolved biogeographical boundaries. The shortcoming of the mentioned studies is that they only consider one species per genus, which is insufficient for genera as diverse as *Clinopodium*. The estimation of the ancestral distribution area also showed that *C. vulgare* is not found in the same clade as the taxa from the American continent, *C. douglasi* and *C. taxifolium*, but is their sister taxon [25]. Moreover, one of the main problems in delimiting the genus is the relationship between *Clinopodium* and *Ziziphora*.

An earlier study included higher species diversity [26]. According to the *trn*L-*trn*F dataset, *C. vulgare* formed a clade with New World taxa, including *Bystropogon* L’Hér. (endemic to the Macaronesian islands). Also, former *Calamintha*, *Acinos*, and *Ziziphora* presented sister taxa to the mentioned clade. This implied that *C. vulgare* is more related to New World than to Old World taxa (*Calamintha*, *Acinos*, and *Ziziphora*). On the other hand, the same research based on ITS analysis showed that *C. vulgare*, *Acinos*, *Ziziphora*, and tree *Bystropogon* taxa form a clade, while *Acinos* and *Ziziphora* were the most related. A big step forward in resolving the genus delimitation and biogeographical implications was a study by Bräuchler et al. [21]. According to this research, the “*Acinos/Ziziphora*” group was supported according to both nrDNA and cpDNA sequences. Some of the taxa in the study, *C. nanum* (P.H. Davis and Doroszenko) Govaerts, *C. troodi* (Post) Govaerts, and *C. rotundifolium*, intercalated with the *Ziziphora* taxa. Considering the obtained results, the authors suggested possible ways for systematics “to keep the lineage separate under one generic name or include it in *Clinopodium*”. Melnikov [27] proposed a new nomenclature based on these results and morphological data. He proposed lumping the genera *Acinos* and *Ziziphora*, forming the subgenus *Acinos* under the genus *Ziziphora*. Thus, led by Bräuchler’s study [21], he transferred former *Acinos* taxa, which were shown as very close to other *Ziziphora* taxa, under the genus *Ziziphora*, subgenus *Acinos* [27]. Still, in online plant databases such as POWO or IPNI (cited previously in the Introduction), the accepted names for these taxa, as well as for former *Acinos* taxa from the Balkans, can be found under the name *Clinopodium*. The names under the genus *Ziziphora* are considered homotypic synonyms, while the name *Clinopodium* continues to take precedence.

However, it is important to note that the *Acinos* taxa distributed in the Balkans formed their own subclade without any *Ziziphora* taxon [21]. These taxa seem to present a well-separated clade with clearly different biology from *Ziziphora*. According to our research, the cpDNA dataset also showed *Z. capitata* as a sister taxon to all other former *Acinos* taxa (Figure 2). In this tree, *Acinos* taxa formed a subclade, but the delimitation at the species and subspecies levels based on the previous phenetic system has not yet been achieved among the former *Acinos* taxa (in the following text: Acinos group). It is important to note that remarkable morphological variability was observed both within and among the populations of the Acinos group. Also, during field research in North Macedonia in 2021, our group recorded several subspecies of former *Acinos* taxa in just one square meter, where the individuals grew almost intertwined. In such a close sympatry, individuals of *C. alpinum*, *C. alpinum* subsp. *meridionale*, and *C. alpinum* subsp. *hungaricum*, along with some morphologically transitional forms, were recorded. The morphological variability mentioned above, supported by such results based on cpDNA sets, increasingly justifies the view of some classic botanists that some species of the former genus *Acinos* represent an aggregate rather than a well-defined, polymorphic species and subspecies [8].

Although the cpDNA tree showed separation between *Acinos* and *Ziziphora*, the concatenated tree and tree based on nrDNA datasets differed slightly, and it showed a similar problem as in the study of Bräuchler [21]. What both cladograms have in common is that *C. alpinum* forms its own subclade, which makes it a sister taxon to all other taxa. In the concatenated tree, *C. alpinum* subsp. *albanicum* is together in this subclade, but in the nrDNA tree, this taxon is in its own clade (cf. Figure 1 and Figure 3). The subspecies within *C. alpinum* are not closely grouped, which is particularly evident in the nrDNA cladogram.

The infraspecific delimitation of the earlier *Acinos* was also not supported by the phylogenetic tree. If we add to these results the observed transitional forms in the habitat and the very close sympatry, there is most likely common hybridisation within this complex. This intertwining of former *Acinos* taxa with *Ziziphora* requires a more detailed study of this complex, considering the variability between and within populations. The premise of this study was that *Ziziphora capitata* should be the only additional outgroup taxon (for this reason, there was only one species of *Ziziphora*). However, the results showed that it is grouped with the taxa of the Acinos group. A recent study of *Z. clinopodioides* Lam. from Iran that included morphological, phytochemical, and molecular (SRAP) markers showed high variability in this taxon, similar to taxa from the Acinos group [28].

### 3.2. Clinopodium–Pseudomelissa–Calamintha Clade

The second well-supported clade in all the analysed datasets is the *Clinopodium*–*Pseudomelissa*–*Calamintha* clade. The previous study also showed the segregation of the taxa from the former genera *Calamintha* and *Micromeria* (section *Pseudomelissa*) with *C. vulgare* [21]. Thus, the authors named this clade *Clinopodium s.s.*, and based on the *trn*K and *trn*L-*trn*F sequences, the taxa from the mentioned group overlapped. According to our results, the analysis restricted only to the cpDNA set did not show separation between former groups on this clade. Bayesian analysis revealed two subclades of mixed representatives, with no apparent differences between them. This overlap of taxa on the cpDNA set is not overly surprising, considering that some botanists have barely distinguished the three groups mentioned based on their morphology [5,13].

On the other side, the concatenated tree and nrDNA tree (Figure 1 and Figure 3) resulted in separate subclades that are entirely congruent with the previous classical systematics of this group [7,8]. In the *Clinopodium*–*Pseudomelissa*–*Calamintha* clade, *C. vulgare* formed its own subclade. Previous *Pseudomelissa* and *Calamintha* taxa were also well supported as separated subclades in the present study. Furthermore, in the concatenated tree, a separation within the former genus *Calamintha* into two subclades was achieved, which also corresponds to the phenetics (*C. menthifolium* and *C. vardarense* formed one subclade, and another subclade carries *C. nepeta* and its subspecies *C. nepeta* subsp. *spruneri*). A good separation was also achieved within the former *Pseudomelissa* group. *Clinopodium pulegium* and *C. dalmaticum* formed a subclade in this clade, and *C. album* was a sister taxon. Based on the AFLP analysis of the *Pseudomelissa* group [29], *C. album* (referred therein as *C. thymifolium*) was most related to *C. dalmaticum*, while *C. pulegium* was a sister to both. This discrepancy on the species level, though low, could be due to the use of individuals of *C. pulegium* from two very isolated populations (the ilyric vs. the moesic region in the present study) that might have been separated from each other for a significant amount of time. Furthermore, the AFLP markers are better suited for the delimitation between closely related species, and perhaps the results of Kremer et al. [29] should be favoured when it comes to relationships within the section *Pseudomelissa*.

In light of the current results, the *Clinopodium*–*Pseudomelissa*–*Calamintha* clade descends from a common ancestor, and all taxa evolved in a relatively short timeframe. The analysis of cpDNA, suitable for relatively deep divergence histories, has led us to this conclusion. The three well-supported subclades, based on their nuclear genomes, indicate that this group went through an intense process of diversification into three lineages, with *C. vulgare* having separated first. This deep separation raised the question of whether this group of taxa represents a clearly defined, unique genus.

### 3.3. General Discussion on Taxonomic Position of Clinopodium Taxa from the Balkans

In the mentioned study by Kremer et al. [29], the strong separation between two previous sections of the genus *Micromeria* was confirmed, viz., between the *Micromeria s.s.* and other taxa from the former section *Pseudomelissa*. The authors note, however, that it is questionable whether the differences are sufficient to characterise these groups as separate genera. They pondered the following question: Should the other groups within *Micromeria* also be separate genera if *Pseudomelissa* is transferred? We would like to pick up here with similar considerations for the genus *Clinopodium*. Our study has confirmed once again that *Pseudomelissa* is a group closer to *C. vulgare* than to *M. croatica* (a representative of *Micromeria s.str.*). But does this group also fit into *Clinopodium* if *Clinopodium* includes species like those from the *Acinos* and *Ziziphora* groups? All the obtained phylogenetic trees show a clear separation of two subclades: the *Clinopodium*–*Pseudomelissa*–*Calamintha* clade and the *Acinos*–*Ziziphora* clade. This separation reflects the different biology and evolutionary history of these clades.

We, therefore, mostly agree with the concept proposed by Melnikov [27] for the taxa *Acinos* and *Ziziphora*, as explained in the first part of the discussion. The current concept of the genus *Clinopodium* is an example of where the best solution could be achieved if we combine current knowledge of phenetics and molecular systematics. The importance of this approach is illustrated by the work of Duminil and Di Michele [30]. The two lineages of *Clinopodium* mentioned above, distributed in the Balkans, showed not only molecular but also different phenetic patterns. The taxa of the former genus *Acinos* are certainly more closely related to *Ziziphora* than to taxa from the second lineage marked as *Clinopodium*. It is of high priority to conduct molecular analyses of the genera *Acinos* and *Ziziphora* at the interpopulation and intrapopulation levels in the near future. However, the taxa of the *Acinos*–*Ziziphora* clade certainly differ from the taxa of the other clades in their life cycle (as mostly annual plants), morphology, and molecular level. According to the results of the present study, it can be presumed that taxa from this study included in the *Acinos*–*Ziziphora* clade should be transferred from *Clinopodium*. For the time being, these results support Melnikov’s, which seems satisfactory. This would mean that the Acinos group should be transferred to *Ziziphora* and thus separated from *Clinopodium*. This could be a step towards a clearer concept of the genus.

This study brought new information regarding the phylogeny within the *Clinopodium s.s.* group proposed by Bräuchler [21], which is referred to as the *Clinopodium*–*Pseudomelissa*–*Calamintha* clade in our study. Nuclear markers have shown that the groups within the clade have evolved separately from each other, which is consistent with phenetic classification mostly based on Bentham’s view. According to Bentham, the section *Pseudomelissa* was considered a natural, separate group, but without distinctive features for the recognition of this group from *Micromeria* as a single genus, or for the other two sections in *Micromeria* [5]. About a decade later, Bentham [31] designated the section *Pseudomelissa* as a transitional group between *Micromeria* and *Calamintha*, comprising nine taxa in this group, including *M. dalmatica*, *M. pulegium*, and *M. rupestris* (now under the name *C. album*), which are included in this study. Bentham believed that the taxa from *Pseudomelissa* belong to *Micromeria* rather than *Clinopodium* due to the distinctive sepals. It is important to note that Bentham [31] divided his heterogeneous genus *Calamintha* into five sections and separated *Clinopodium* from *Calamintha*. *C. vulgare* was placed in the section *Clinopodium* (under the name *Clinopodium clinopodium*). According to our results based on nrDNA, the clades and subclades obtained are fully consistent with classical taxonomy (*Clinopodium—C. vulgare*, *Calamintha*, and *Pseudomelissa* are separate groups, i.e., separate subclades). However, Bräuchler [21] transferred taxa from *Pseudomelissa* to *Clinopodium* and pointed out that the notched margin, lack of marginal vein, and chromosome number are more characteristic of *Clinopodium* than of taxa from *Micromeria*. He also pointed out that the taxa of *Pseudomelissa* have almost the same *trn*K and *trn*L-*trn*F regions, but according to the presented cladogram, the taxa of *Pseudomelissa* are actually on the sister subclade to the clade that carries *C. vulgare*. Obviously, the cpDNA set has some limitations in reconstructing the phylogenetic relationships of this group. Again, the question arises as to which marker should be favoured, but again, we would conclude that the broader context for each plant group should be considered. Although there are reasons to consider the former genera *Calamintha* and *Pseudomelissa* as *Clinopodium*, having separated these three groups based on nrDNA and morphological distinguishing characters, we believe that these groups have the potential to be separated taxonomically. To clarify the relationships in this clade, a broader sample of taxa is required (more species diversity and more populations per taxon). In addition, phylogeographic and phenotypic analyses should be combined to obtain the most reliable results. A comprehensive morphological analysis of this group is underway.

## 4. Materials and Methods

### 4.1. Taxon Sampling

The taxa were chosen to cover most of the diversity of *Clinopodium* in the Balkan Peninsula. Thus, we included in this study the representatives of the former genera: *Acinos* Mill., *Calamintha* Mill., *Micromeria* Benth. (section *Pseudomelissa* Benth.), and *Clinopodium vulgare* L., as typical representatives of the genus (cf. Table 2). The outgroup taxa were chosen according to the previous phylogenetic studies, which concerned group Menthinae [11,13,21]. Considering this, we chose *Micromeria croatica* (Pers.) Schott as a representative of the *Micromeria s.s.* group, which is supported as sufficiently distanced from *Clinopodium* and belonging to the genus *Micromeria*. The selection of *M. croatica* as an outgroup taxon resulted from the fact that part of the genus *Micromeria* was transferred to *Clinopodium*, whereas *M. croatica* had always been positioned in the group *Micromeria s.s.* The other outgroup taxon was *Ziziphora capitata* L., which formed the “*Acinos*”*/Ziziphora* group together with the former *Acinos* taxa [21]. The greatest diversity of *Ziziphora* species is found mainly in the Irano-Turanian floristic region. In the Balkans, the diversity drops, and to the best of our knowledge, there is only the species *Z. capitata*, which we have included in this study. We only considered *Z. capitata* as a species from the Balkans that is sympatric with the other taxa analysed. Due to the scope of this research, we included only taxa from the Balkans, since hybridisation and introgression in Lamiaceae are too common.

In the field, leaves of each analysed individual were packed in sterile filter bags and desiccated using silica gel. The voucher specimens of all the analysed populations were deposited in the BEOU (Herbarium of the University of Belgrade, Faculty of Biology and Botanical Garden Jevremovac). Details of the collected material are given in Table 2.

### 4.2. Choice of Molecular Markers

The selection of the markers was preceded by an extensive in silico analysis of the available chloroplast regions in the NCBI database. The informativity of the regions was queried by analysing the sequences of the three species from the *Satureja s.l.* group that are available in the GenBank accessions (*C. abyssinicum* NC_058327.1, *C. chinense* NC_050943.1, *Satureja montana* NC_066034.1). The in silico analysis included 43 chloroplast intergenic spacers (*acc*D-*psa*I, *atp*B-*rbc*L, *rpl*32-*trn*L^UAG^, *atp*F-*atp*H, *atp*I-*atp*H, *trn*L^UAA^-*trn*F^GAA^, *ccs*A-*ndh*D, *clp*P-*psb*B, *ndh*C-*trn*V^UAC^, *ndh*E-*ndh*I, *pet*A-*psb*J, *pet*B-*pet*D, *pet*L-*psa*J, *pet*N-*psb*M, *psa*A-*ycf*3, *psa*C-*ndh*E, *psa*I-*ycf*4, *psa*J-*rpl*20, *psb*A-*trn*H^GUG^, *psb*K-*trn*S^UGA^, *rbc*L-*acc*D, *rpl*20-*rps*12, *rpl*23-*psb*A, *rps*3-*rps*19, *rps*4-*trn*T, *rps*16-*trn*Q^UUG^, *rps*15-*ycf*1, *rps*16-*trn*K^UUU^, *rrn*23-*trn*A, *trn*C-*trn*D, *trn*D-*trn*T, *trn*F-*ndh*J, *trn*F-*psb*A, *trn*G-*atp*A, *trn*H^GUG^-*trn*K^UUU^, *trn*K^UUU^-*mat*K-*trn*K^UUU^, *trn*K^UUU^-*trn*Q^UUG^, *trn*L^UAG^-*rpl*32, *trn*Q^UUG^-*psb*K, *trn*S^UGA^-*psb*Z, *trn*T-*psb*C, *ycf*3-*trn*S^UGA^, and *ycf*4-*ycf*10) and six introns (*atp*F intron, *clp*P introns 1 and 2, *rpoC*1 intron, *trn*V intron, and *ycf*3 intron 1). The regions with the highest base substitutions and microsatellite loci were selected. In total, three nrDNA spacers (ITS1, ITS2, ETS), a coding region of 5.8S rDNA, and seven chloroplast intergenic spacers (*rps*16-*trn*K^UUU^, *rpl*32-*trn*L^UAG^, *rps*15-*ycf*1, *psb*A-*trn*H^GUG^, *rps*16-*trn*Q^UUG^, *pet*N-*psb*M, *psb*K-*trn*S^UGA^) were chosen for experimental analysis.

### 4.3. DNA Extraction, Amplification, and Sequencing

DNA was extracted from the young leaves of sixteen individuals using the CTAB protocol [32], with modifications previously detailed in [33,34]. DNA purity and quantity were assessed with the Nanophotometer N60/N50 NanoVolume (IMPLEN, München, Germany). All samples were diluted to 100 ng/µL and preserved at −70 °C until the PCR reactions.

The PCR products were obtained using the primers listed in Table 1. PCR amplifications were conducted in a volume of 25 μL containing 50–100 ng of DNA at a specified concentration. The master mix for each sample was prepared to include 1 × buffer A (Nippon Genetics Europe, Düren, Germany); 1 mM MgCl2; 0.2 mM dNTPs; 0.25 μM of each forward (F) and reverse (R) primer; 0.2 μL of BSA (bovine serum albumin); and 1U of FastGene^®^ Taq DNA Polymerase (Nippon Genetics Europe).

The following PCR amplification program was used: initial denaturation at 94 °C for 5 min; 35 cycles of denaturation at 94 °C for 60 s; annealing for 60 s at 51–63 °C; extension at 72 °C for 45–90 s (c.f. Table 3); and a final extension of 10 min at 72 °C. After completing the PCR program, the results of each reaction were checked by electrophoresis.

PCR products were sequenced by Macrogen Europe (Amsterdam, The Netherlands) via Sanger sequencing using a 96-capillary 3730xl DNA analyser automated sequencer (Applied Biosystems, Waltham, MA, USA).

### 4.4. Phylogenetic Analyses

Sequence chromatograms were manually checked, edited, and aligned using MEGA 11 [40]. All sequences obtained in this research were deposited in GenBank (Supporting Information, Table S1). The sequences were easily alignable among all the accessions in all the plastid and nuclear matrices. Phylogenetic analyses were conducted using maximum likelihood (ML) and Bayesian inference (BI) algorithms applied to individual loci, concatenated plastid and nuclear loci separately, and as a concatenated matrix using all data. Gaps in the alignment were treated as missing data. *Micromeria croatica* and *Ziziphora capitata* were used as outgroups for all analyses. 

The ML analyses were conducted using RaxML 8.0 [41] alongside the raxmlGUI v. 2.0.13 [42], adhering to the default parameter settings. The evolutionary models for each locus were calculated with Modetl-Test-NG [43]. Statistical support for the nodes was determined based on 100 non-parametric bootstrap replicates (BS), with values of ≥75% regarded as good support. 

The BI analyses were conducted with BEAST v2.7.7 [44]. The input file for the BEAST analyses was constructed using the BEAUti interface of the BEAST package, and the file with parameter settings was executed in BEAST [45]. We used a GTR+G model with four categories of rate heterogeneity for the final analysis and a demographic model of constant population size as a tree before modelling changes in population size through time [46,47]. Following a burn-in of 1 million steps, all parameters were sampled once every 1000 steps from 5 million MCMC steps. TreeAnnotator 2.7.6 (part of the BEAST package) was used to construct a majority-rule consensus tree using the trees remaining after the burn-in and also to summarise the posterior distributions of nodes. For the BI analyses, posterior probabilities (PP) ≥ 0.98 were considered good support. Trees were visualised and edited using Figtree 1.4.4 (https://tree.bio.ed.ac.uk/software/figtree/, accessed on 11 January 2025). All trees were rooted using *Micromeria croatica*. 

## 5. Conclusions

This study is dedicated to deepening the understanding of the relationships within the heterogeneous genus *Clinopodium*, considering taxa with a distribution range in the Balkans. Although the analysed group represents a relatively small group of the genus *Clinopodium*, this is important in view of the geographical distribution and heterogeneity of the species and the use of highly variable sequences. Almost all molecular markers were used for the first time in the phylogeny of these taxa. Two phylogenetic lineages, well supported in all trees, were determined: *Acinos*/*Ziziphora* and *Clinopodium*–*Pseudomelissa*–*Calamintha* clades. These two lineages showed different patterns of diversification. In the *Acinos*/*Ziziphora* clade, these two genera appear to have been well defined in the past (based on the cpDNA set), with intense introgression in more recent times (nrDNA). In the clade *Clinopodium*–*Pseudomelissa*–*Calamintha*, however, the opposite evolutionary path is assumed, with all subclades representing a clear lineage without hybridisation between the subclades. The earlier phylogenetic results, where the taxa were analysed on a broader scale, have shown that all these taxa are very closely related. The combined set of highly informative molecular markers allowed a finer resolution of the relationships between taxa that belong to separate genera based on phenetics. This approach contributes to the consideration of the *Clinopodium* concept and future taxonomic treatment. The question is whether this dichotomy can underlie a single generic affiliation. We propose the transfer of *Acinos* taxa from *Clinopodium* to the genus *Ziziphora*. At this stage, it seems appropriate to treat *Clinopodium*–*Pseudomelissa*–*Calamintha* as *Clinopodium s.s.* However, this group needs further in-depth investigation to understand the phylogenetic relationships better and determine its taxonomic treatment as well as biogeographical patterns.

## Figures and Tables

**Table 1:** The informativeness of the analysed regions.

Locus	Length ^1^	No. of Indels ^2^	Indel Length in bp ^3^	Substitutions		Microsatellite Loci
				Transitions	Transversions	
*trn*S^UGA^-*psb*K	703	21	90	11	19	13
*trn*L^UAG^-*rpl*32	903	16	91	27	34	34
*rps*16-*trn*Q^UUG^	1002	22	206	21	31	41
*rps*16*-trn*K^UUU^	710	15	67	19	23	18
*rps*15-*ycf*1	611	12	144	24	12	19
*psb*A-*trn*H^GUG^	424	10	61	13	21	13
*pet*N-*psb*M	1057	19	47	21	17	25
ITS	702	15	21	77	47	17
ETS	408	16	24	138	68	8

^1^ The aligned sequence length for each locus, exported directly from MEGA. ^2^ The indels in each sequence are variable in length. ^3^ Bp—base pairs. Indels and substitutions are generated manually. Microsatellites were detected using Microsatellite Repeats Finder (http://insilico.ehu.es/mini_tools/microsatellites/, accessed on 16 January 2025).

**Table 2:** Details of the collected material, including accepted taxon name, locality and herbarium number.

Taxon	Locality	Long.	Lat.	Alt [m a.s.l.]	BEOU
Former *Acinos* taxa					
*Clinopodium acinos* (L.) Kuntze	Serbia, Mt. Tara	43.865	19.406	872	17,987
*Clinopodium alpinum* (L.) Kuntze subsp. *alpinum*	Serbia, Topli Do	43.334	22.664	690	17,990
*Clinopodium alpinum* subsp. *albanicum* (Kümmerle & Jáv.) Govaerts	Serbia, Mt. Rogozna	43.045	20.521	882	17,992
*Clinopodium alpinum subsp. hungaricum* (Simonk.) Govaerts	Serbia, Mt. Fruska gora	45.156	19.778	387	17,993
*Clinopodium alpinum* subsp. *majoranifolium* (Mill.) Govaerts	Montenegro, Kotor	42.423	18.791	534	18,037
*Clinopodium suaveolens* (Sm.) Kuntze	Serbia, Mt. Rtanj	43.767	21.926	791	18,001
Former *Calamintha* taxa					
*Clinopodium menthifolium* (Host.) Merino	Serbia, Mt. Tara	43.969	19.344	1010	18,039
*Clinopodium vardarense* (Šilić) Govaerts	N. Macedonia, Stenje	40.934	20.930	818	18,042
*Clinopodium nepeta* subsp. *spruneri* (Boiss.) Bartolucci & F.Conti	Croatia, Vrana lake	43.848	15.636	9	18,041
*Clinopodium nepeta* (L.) Kuntze	Serbia, Belgrade	44.815	20.472	143	18,040
Former *Pseudomelissa* taxa					
*Clinopodium album* (Waldst. & Kit.) Bräuchler & Govaerts	Serbia, Mt. Tara	43.866	19.407	872	18,004
*Clinopodium pulegium* (Rochel) Bräuchler	Serbia, Svrljig gorge	43.542	22.177	259	17,999
*Clinopodium dalmaticum* (Benth.) Bräuchler & Heubl	Montenegro, Njeguši	42.408	18.787	912	18,038
*Clinopodium* L.					
*Clinopodium vulgare* L.	Serbia, Zlot	44.029	21.961	303	18,043
Outgroup taxa					
*Ziziphora capitata* L.	Serbia, Svrljig gorge	43.542	22.177	259	18,045
*Micromeria croatica (Pers.) Schott*	Serbia, Mt. Tara	43.864	19.409	844	18,044

**Table 3:** Chloroplast and nuclear loci used in this study.

Locus		Primer Sequence (Seq 5′-3′)	Ta [°C]	Ext. [s]	Reference
*rps*16-*trn*K^UUU^	F R	TTAAAAGCCGAGTACTCTACC AAAGTGGGTTTTTATGATCC	53	60	[35]
*rpl*32-*trn*L^UAG^	F R	CAGTTCCAAAAAAACGTACTTC CTGCTTCCTAAGAGCAGCGT	53	60	[35]
*rps*15-*ycf*1	F R	CAATTYCAAATGTGAAGTAAGTCTCC CTTGTATGRATCGTTATTGKTTTG	58	60	[35]
*psb*A-*trn*H^GUG^	F R	GTTATGCATGAACGTAATGCTC CGCGCATGGTGGATTCACAATCC	53	60	[36]
*rps*16-*trnK*^UUU^	F R	GTTTCAAACGAAGTTTTACCAT TCGAATCCTTCCGTCCC	51	75	[37]
*pet*N-*psb*M	F R	ATGGATATAGTAAGTCTCGCTTG ATGGAAGTAAATATTCTTGCAT	51	90	[37]
*psb*K-*trn*S^UGA^	F R	TTTGGCAGGCTGCTGTAAGTT ACTAAAGCGTCGGATTGCT	56.5	60	*
ITS	F R	GGAAGTAAAAGTCGTAACAAGG TCCTCCGCTTATTGATATGC	63	45	[38]
ITS	F R	GTCCACTGAACCTTATCATTTAG TCCTCCGCTTATTGATATGC	55	60	[21]
ETS	F R	GTGAGTGGTGKTTGGCGYGT GCAGGATCAACCAGGTAGCA	55	60	[39]

Ta—annealing temperature; Ext—extension time within the cycle. For the ITS region, an alternative primer pair was used when no product was obtained. * Designed by the authors of the present work.

## Data Availability

The original contributions presented in this study are included in the article/Supplementary Material. Further inquiries can be directed to the corresponding author.

## Supplementary Materials

The following supporting information can be downloaded at https://www.mdpi.com/article/10.3390/plants14182940/s1: Table S1: NCBI accession numbers of sequences obtained in this study.
